# Determinants of Shielding Behavior During the COVID-19 Pandemic and Associations With Well-being Among National Health Service Patients: Longitudinal Observational Study

**DOI:** 10.2196/30460

**Published:** 2021-09-20

**Authors:** Patrik Bachtiger, Alexander Adamson, William A Maclean, Mihir A Kelshiker, Jennifer K Quint, Nicholas S Peters

**Affiliations:** 1 National Heart and Lung Institute Imperial College London London United Kingdom; 2 Imperial College Healthcare NHS Trust London United Kingdom

**Keywords:** COVID-19, shielding, well-being, personal health record, determinant, behavior, protection, longitudinal, observational, health policy, mental health, epidemiology, public health

## Abstract

**Background:**

The UK National Health Service (NHS) classified 2.2 million people as clinically extremely vulnerable (CEV) during the first wave of the 2020 COVID-19 pandemic, advising them to “shield” (to not leave home for any reason).

**Objective:**

The aim of this study was to measure the determinants of shielding behavior and associations with well-being in a large NHS patient population for informing future health policy.

**Methods:**

Patients contributing to an ongoing longitudinal participatory epidemiology study (Longitudinal Effects on Wellbeing of the COVID-19 Pandemic [LoC-19], n=42,924) received weekly email invitations to complete questionnaires (17-week shielding period starting April 9, 2020) within their NHS personal electronic health record. Question items focused on well-being. Participants were stratified into four groups by self-reported CEV status (qualifying condition) and adoption of shielding behavior (baselined at week 1 or 2). The distribution of CEV criteria was reported alongside situational variables and univariable and multivariable logistic regression. Longitudinal trends in physical and mental well-being were displayed graphically. Free-text responses reporting variables impacting well-being were semiquantified using natural language processing. In the lead up to a second national lockdown (October 23, 2020), a follow-up questionnaire evaluated subjective concern if further shielding was advised.

**Results:**

The study included 7240 participants. In the CEV group (n=2391), 1133 (47.3%) assumed shielding behavior at baseline, compared with 633 (13.0%) in the non-CEV group (n=4849). CEV participants who shielded were more likely to be Asian (odds ratio [OR] 2.02, 95% CI 1.49-2.76), female (OR 1.24, 95% CI 1.05-1.45), older (OR per year increase 1.01, 95% CI 1.00-1.02), living in a home with an outdoor space (OR 1.34, 95% CI 1.06-1.70) or three to four other inhabitants (three: OR 1.49, 95% CI 1.15-1.94; four: OR 1.49, 95% CI 1.10-2.01), or solid organ transplant recipients (OR 2.85, 95% CI 2.18-3.77), or have severe chronic lung disease (OR 1.63, 95% CI 1.30-2.04). Receipt of a government letter advising shielding was reported in 1115 (46.6%) CEV participants and 180 (3.7%) non-CEV participants, and was associated with adopting shielding behavior (OR 3.34, 95% CI 2.82-3.95 and OR 2.88, 95% CI 2.04-3.99, respectively). In CEV participants, shielding at baseline was associated with a lower rating of mental well-being and physical well-being. Similar results were found for non-CEV participants. Concern for well-being if future shielding was required was most prevalent among CEV participants who had originally shielded.

**Conclusions:**

Future health policy must balance the potential protection from COVID-19 against our findings that shielding negatively impacted well-being and was adopted in many in whom it was not indicated and variably in whom it was indicated. This therefore also requires clearer public health messaging and support for well-being if shielding is to be advised in future pandemic scenarios.

## Introduction

At the start of the first wave of the COVID-19 pandemic, the UK National Health Service (NHS) identified 2.2 million people as clinically extremely vulnerable (CEV), and they were assumed to be at high risk of severe COVID-19 infection [1]. CEV status was conferred by the severity, history, and treatment levels of specific conditions [2]. These individuals were notified by a postal letter (March 25, 2020, onwards) to enter an unprecedented period of shielding; a voluntary action that, at the time, instructed “stay at home at all times and avoid all face-to-face contact for at least 12 weeks” [3]. CEV individuals were also advised to practice social distancing with those in the household not required to shield and to only have in-person encounters for health or social care reasons.

Whether the action of shielding reduced the risk of exposure to COVID-19 in the UK is particularly difficult to measure for the first wave, given largely absent at-home or community testing [4,5]. Acknowledging the potentially negative impact the original policy was having on general well-being, the shielding policy was revised after 4 months, such that CEV patients could, if they wished, meet as a group of up to six people outdoors and form a “support bubble” with another household (July 6, 2020, onwards) [6]. Ubiquitous government messaging had by then established the notion of “shielding” among health care professionals and the wider British public [7]. The prevalence of shielding behavior among patients in whom it is not indicated and its potential harm remain unknown. More broadly, there is inadequate understanding of the determinants for adopting shielding behavior or the potential trade-off in well-being when doing so [8].

Therefore, the aim of our study was to explore the determinants of adopting shielding behavior and its relationship with well-being. Our study is informed by the likelihood that (1) shielding behavior is likely to impact well-being, and (2) the decision to shield is influenced by the anticipated impacts on well-being, with implications for adherence to public health advice. Our study specifically examines how demographic and lifestyle factors may impact both the choices to shield and associated outcomes of shielding. The intention was to test a hypothesis that shielding behavior in the first wave was determined by variables beyond CEV status and was overall detrimental to well-being.

## Methods

### Study Design

**Figure 1:**
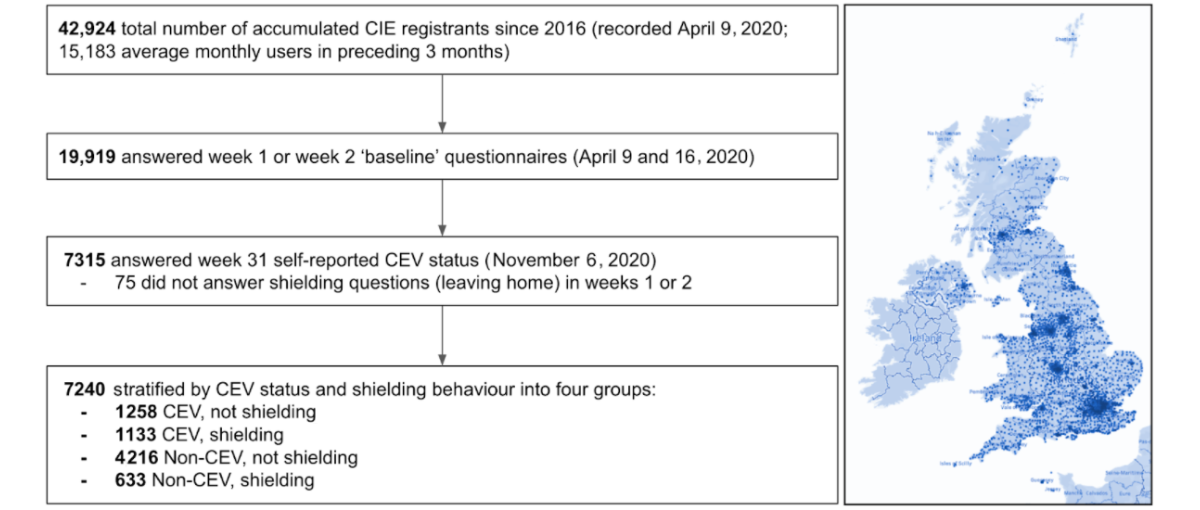
Study inclusion diagram. Left: participant selection and stratification based on responses to baseline (week 1 and 2) and CEV status (week 31) questionnaires. Right: map of CIE registrants by UK postcode. CEV: clinically extremely vulnerable; CIE: Care Information Exchange.

The Longitudinal Effects on Wellbeing of the COVID-19 Pandemic (LoC-19) study began inviting registrants of the Care Information Exchange (CIE) (Imperial College Healthcare NHS Trust, UK) to complete a weekly questionnaire as a direct care tool for self-monitoring their well-being, starting (“week 1”) April 9, 2020. The CIE is the NHS’s largest patient personal electronic health record with UK-wide registrants (Figure 1). Patients can access their digital health records within the CIE after an index encounter (eg, blood test, outpatient appointment, and inpatient admission) triggers creation of a record. Starting from 2016, on April 9, 2020, the CIE had accumulated 42,924 registrants, with a mean average number of 15,183 monthly active users (defined by at least one login) in the 3 months preceding the beginning of the LoC-19 study. LoC-19 participants would receive a weekly email notification to complete questionnaires within their CIE record, where responses were retained as a direct care tool for self-monitoring well-being during the pandemic. The subsequent online community of participants was also provided with regular feedback on how responses were being used to also inform local and national health policy.

### Questionnaire Items and Outcome Measures

Applying recommendations for questionnaire design [9,10], question items were developed by a collaboration of experts in qualitative research at Imperial College London, encompassing public health, respiratory epidemiology, and digital health, and were also informed by previous studies [11,12]. Question items were externally peer reviewed and tested on lay persons (n=5) before being included. The themes covered included mental and physical well-being, situational variables (eg, home setting), and COVID-19 testing and symptoms. Receipt of government letters advising shielding (weeks 1 and 2) and CEV status (week 31, November 6, 2020, to capture the latest additions to the CEV list after week 2) were also recorded. Due to the weekly nature of the questionnaires, it was important to minimize bias introduced by respondent fatigue [13]; therefore, question items for mood and well-being were simplified to a Likert scale (1 to 10). Participants were also asked (week 14, July 10, 2020) to submit free-text responses to a question on the most difficult aspect of lockdown. A further question item posed in a separate questionnaire (week 29, October 23, 2020) evaluated prospective attitudes toward the need to assume shielding in the face of a further national lockdown. The questionnaires sent out in weeks 1 and 2 were almost identical and were used to define the baseline population. If participants answered both weeks, week 1 responses were used to baseline shielding behavior. A detailed description of variables captured by questionnaires used in this study is presented in Multimedia Appendix 1.

### Ethical Approval

The weekly questionnaire was a direct care tool for patients to self-monitor their well-being during the COVID-19 pandemic. Participants were not paid or otherwise compensated for completing questionnaires. Upon review, the Imperial College Healthcare NHS Trust Data Protection Office advised that ethical approval for data analysis and publication was not required. Participants gave informed consent within the CIE, were free to opt out of receiving questionnaires at any time, and were informed prior to completing their responses that these would be fully anonymized and stored securely.

### Participant Selection

We considered participants aged 18 years or above and those who answered questionnaires relevant to the analysis (Figure 1). Responses submitted later than 4 days from the release day of each weekly questionnaire were excluded.

### Stratification of Study Groups

To align with the government’s official definition of shielding behavior, participants who exclusively answered “I am not leaving my home” in response to what activity they were leaving home for (eg, “commute to work,” “essential shopping,” “exercise,” and “other”) were categorized as exhibiting shielding behavior. Classification of CEV or non-CEV status was defined by participants’ responses to a question item listing clinical conditions conferring CEV status, which included being given clinical advice to shield. This classification gave rise to the following four participant groups: CEV and not shielding, CEV and shielding, non-CEV and not shielding, and non-CEV and shielding.

### Data Analysis

Shielding behavior was compared within CEV and non-CEV groups separately, as these two patient groups were characteristically different, and it was not deemed appropriate to combine them. Groups were stratified in this way rather than through the use of an interaction term to ease interpretability of the analysis. Persistent shielding was defined as shielding at baseline, week 9, and week 15.

Descriptive statistics are reported alongside odds ratios (ORs) from univariable and multivariable logistic regression for the association with shielding behavior. ORs will not approximate relative risks as the outcome becomes more common and as ORs diverge further from 1. Variables included in the multivariable regression were selected a priori for their clinical relevance and to avoid multicollinearity from similar explanatory variables. Differences in categorical variables were assessed by chi-square tests or Fisher exact tests where chi-square test assumptions were violated, and differences in continuous variables were assessed using *t* tests. *P* values <.05 were considered statistically significant.

The 10-point scale measurements of “how do you feel today” in relation to “physically” and “mood” (1=worst, 10=best) were assessed using linear mixed effects regression, with “mood” and “physically” included as continuous outcome variables and patient included as the clustering variable. Week was centered and included as an explanatory variable in the model with a quadratic term. Week was included as a random effect, with the effect of week on the outcome allowed to vary by patient. The null model included week in the model only, univariable analyses added shielding into the model as an explanatory variable, and multivariable analyses added the same variables used when assessing associations with shielding behavior. Differences across time for each group are represented by mean values for each participant group on a longitudinal plot across all weeks.

Free-text responses (n=6300) were labeled in a multilabel supervised machine learning setting for efficiency. Responses were vectorized, and a logistic regression model was trained on 3000 manually labeled responses. Three researchers first independently manually labeled 100 responses each and reached a consensus on categories for labeling the remaining 2700. The remaining 3300 were labeled with this model, and every label was verified by sight and altered if necessary. Results are displayed as a stacked bar chart. All analyses were carried out in R version 3.6.2 or Python version 3.7.

### Data Sharing

Imperial College Healthcare NHS Trust is the data controller. The data sets analyzed in this study are not publicly available, but can be shared for scientific collaboration subject to meeting the requirements of the institution’s data protection policy.

## Results

### Baseline Characteristics

The study sample included 7240 participants. In the CEV group (n=2391), 1133 (47.3%) assumed shielding behavior at baseline, compared with 633 (13.0%) in the non-CEV group (n=4849). The average number of questionnaires completed by participants did not differ by group, with participants completing an average 75% of the first 17 weekly questionnaires. Baseline characteristics and ORs from univariable and multivariable analyses are shown for the four groups in Table 1. Similarly, information for situational variables is presented in Table 2, and information for qualifying CEV status distribution is presented in Table 3. Among those initially shielding, 29.0% (329/1133) in the CEV group and 17.7% (112/633) in the non-CEV group were still doing so at week 15.

**Table 1:** Patient characteristics and questionnaire responses grouped by baseline demographics.

Characteristic		CEV^a^, not shielding (N=1258)	CEV, shielding (N=1133)	CEV (univariable), OR^b^ (95% CI)	*P* value	CEV (multivariable), OR^b^ (95% CI)	*P* value	Non-CEV, not shielding (N=4216)	Non-CEV, shielding (N=633)	Non-CEV (univariable), OR^b^ (95% CI)	*P* value	Non-CEV (multivariable), OR^b^ (95% CI)	*P* value
Questionnaires completed (out of 17 possible), mean (SD)		12.8 (2.6)	12.7 (2.8)	0.99 (0.96-1.01)	.33	N/A^c^	N/A	12.8 (2.6)	12.6 (2.8)	0.98 (0.95-1.01)	.17	N/A	N/A
Age (years)^d^, mean (SD)		58.5 (13.0)	60.2 (13.3)	1.01 (1.00-1.02)	.002	1.02 (1.01-1.03)	<.001	57.5 (13.7)	60.8 (14.4)	1.02 (1.01-1.03)	<.001	1.02 (1.01-1.03)	<.001
**Sex, n (%)**													
	Male	608 (48.3)	488 (43.1)	N/A	N/A	N/A	N/A	1954 (46.3)	264 (41.7)	N/A	N/A	N/A	N/A
	Female	650 (51.7)	645 (56.9)	1.24 (1.05-1.45)	.01	1.33 (1.09-1.62)	.004	2262 (53.7)	369 (58.3)	1.21 (1.02-1.43)	.03	1.43 (1.18-1.74)	<.001
**Ethnicity, n (%)**													
	White	898 (71.4)	758 (66.9)	N/A	N/A	N/A	N/A	3160 (75.0)	416 (65.7)	N/A	N/A	N/A	N/A
	Asian	72 (5.7)	123 (10.9)	2.02 (1.49-2.76)	<.001	2.10 (1.51-2.95)	<.001	206 (4.9)	68 (10.7)	2.51 (1.86-3.34)	<.001	2.65 (1.94-3.58)	<.001
	Black	48 (3.8)	32 (2.8)	0.79 (0.50-1.24)	.31	0.79 (0.48-1.30)	.37	102 (2.4)	12 (1.9)	0.89 (0.46-1.57)	.72	1.06 (0.53-1.93)	.86
	Mixed	14 (1.1)	6 (0.5)	0.51 (0.18-1.27)	.17	0.43 (0.15-1.15)	.11	59 (1.4)	13 (2.1)	1.67 (0.87-2.98)	.10	2.14 (1.11-3.87)	.02
	Other	56 (4.5)	47 (4.1)	0.99 (0.66-1.48)	.98	1.13 (0.73-1.74)	.57	167 (4.0)	39 (6.2)	1.77 (1.22-2.52)	.002	2.15 (1.45-3.12)	<.001
	Prefer not to say	18 (1.4)	13 (1.1)	0.86 (0.41-1.75)	.67	0.84 (0.38-1.84)	.66	24 (0.6)	7 (1.1)	2.22 (0.88-4.91)	.07	2.81 (1.00-6.80)	.03
	Missing	152 (12.1)	154 (13.6)	N/A	N/A	N/A	N/A	498 (11.8)	78 (12.3)	N/A	N/A	N/A	N/A
**Smoking status, n (%)**													
	Never smoker	707 (56.2)	653 (57.6)	N/A	N/A	N/A	N/A	2512 (59.6)	411 (64.9)	N/A	N/A	N/A	N/A
	Exsmoker	465 (37.0)	417 (36.8)	0.97 (0.82-1.15)	.73	0.95 (0.78-1.17)	.63	1430 (33.9)	195 (30.8)	0.83 (0.69-1.00)	.05	0.83 (0.67-1.01)	.07
	Smoker	82 (6.5)	63 (5.6)	0.83 (0.59-1.17)	.30	0.98 (0.65-1.47)	.92	264 (6.3)	25 (3.9)	0.58 (0.37-0.87)	.01	0.70 (0.43-1.09)	.14
	Missing	4 (0.3)	N/A	N/A	N/A	N/A	N/A	10 (0.2)	2 (0.3)	N/A	N/A	N/A	N/A

^a^CEV: clinically extremely vulnerable.

^b^Odds ratios (ORs) represent the likelihood of adopting shielding behavior, expressed with associated 95% CIs.

^c^N/A: not applicable.

^d^Age showed a linear association with shielding behavior and was included as a linear continuous variable, with ORs representing a +1 increase in yearly age.

**Table 2:** Patient characteristics and questionnaire responses grouped by situational variables.

Situational variable		CEV^a^, not shielding (N=1258)	CEV, shielding (N=1133)	CEV (univariable), OR^b^ (95% CI)	*P* value	CEV (multivariable), OR (95% CI)	*P* value	Non-CEV, not shielding (N=4216)	Non-CEV, shielding (N=633)	Non-CEV (univariable), OR (95% CI)	*P* value	Non-CEV (multivariable), OR (95% CI)	*P* value
**Key worker,** ** n (%)**													
	No	1083 (86.1)	1010 (89.1)	N/A^c^	N/A	N/A	N/A	3641 (86.4)	567 (89.6)	N/A	N/A	N/A	N/A
	Yes	163 (13.0)	112 (9.9)	0.74 (0.57-0.95)	.02	0.74 (0.54-1.01)	.06	556 (13.2)	54 (8.5)	0.62 (0.46-0.83)	.002	0.63 (0.44-0.87)	.007
	Missing	12 (1.0)	11 (1.0)	N/A	N/A	N/A	N/A	19 (0.5)	12 (1.9)	N/A	N/A	N/A	N/A
**Outdoor space, n (%)**													
	No outdoor space	197 (15.7)	136 (12.0)	N/A	N/A	N/A	N/A	696 (16.5)	81 (12.8)	N/A	N/A	N/A	N/A
	Outdoor space	902 (71.7)	834 (73.6)	1.34 (1.06-1.70)	.02	1.20 (0.92-1.57)	.19	3011 (71.4)	470 (74.2)	1.34 (1.05-1.73)	.02	1.33 (1.02-1.76)	.04
	Missing	159 (12.6)	163 (14.4)	N/A	N/A	N/A	N/A	509 (12.1)	82 (13.0)	N/A	N/A	N/A	N/A
**Household members, n (%)**													
	1	320 (25.4)	250 (22.1)	N/A	N/A	N/A	N/A	980 (23.2)	119 (18.8)	N/A	N/A	N/A	N/A
	2	562 (44.7)	480 (42.4)	1.09 (0.89-1.34)	.40	0.87 (0.68-1.10)	.24	1825 (43.3)	293 (46.3)	1.32 (1.06-1.66)	.02	1.25 (0.98-1.61)	.08
	3	175 (13.9)	204 (18.0)	1.49 (1.15-1.94)	.003	1.32 (0.97-1.79)	.08	665 (15.8)	114 (18.0)	1.41 (1.07-1.86)	.01	1.49 (1.09-2.03)	.01
	4	112 (8.9)	130 (11.5)	1.49 (1.10-2.01)	.01	1.33 (0.91-1.93)	.14	479 (11.4)	66 (10.4)	1.13 (0.82-1.56)	.44	1.19 (0.81-1.73)	.37
	5+	76 (6.0)	63 (5.6)	1.06 (0.73-1.54)	.76	1.00 (0.63-1.58)	.99	236 (5.6)	33 (5.2)	1.15 (0.75-1.72)	.50	1.39 (0.85-2.21)	.17
	Missing	13 (1.0)	6 (0.5)	N/A	N/A	N/A	N/A	31 (0.7)	8 (1.3)	N/A	N/A	N/A	N/A
**Letter advising shielding, n (%)**													
	Not received	836 (66.5)	424 (37.4)	N/A	N/A	N/A	N/A	4063 (96.4)	574 (90.7)	N/A	N/A	N/A	N/A
	Received	414 (32.9)	701 (61.9)	3.34 (2.82-3.95)	<.001	3.57 (2.96-4.32)	<.001	128 (3.0)	52 (8.2)	2.88 (2.04-3.99)	<.001	2.73 (1.83-4.00)	<.001
	Missing	8 (0.6)	8 (0.7)	N/A	N/A	N/A	N/A	25 (0.6)	7 (1.1)	N/A	N/A	N/A	N/A
Shielding behavior (week 9), n (%)		159 (14.5)	523 (53.2)	6.66 (5.42-8.24)	<.001	N/A	N/A	129 (3.5)	216 (39.9)	18.33 (14.36-23.49)	<.001	N/A	N/A
Shielding behavior (week 15), n (%)		76 (7.4)	255 (29.0)	5.10 (3.89-6.75)	<.001	N/A	N/A	65 (1.9)	92 (17.7)	11.13 (8.00-15.59)	<.001	N/A	N/A
Any health care utilization, n (%)		1175 (93.4)	1065 (94.0)	1.11 (0.80-1.54)	.55	N/A	N/A	3277 (77.7)	518 (81.8)	1.29 (1.05-1.61)	.02	N/A	N/A
Emergency department attendance, n (%)		111 (8.8)	131 (11.6)	1.35 (1.04-1.77)	.03	N/A	N/A	304 (7.2)	52 (8.2)	1.15 (0.84-1.55)	.37	N/A	N/A
GP^d^ in-person consultation, n (%)		428 (34.0)	376 (33.2)	0.96 (0.81-1.14)	.67	N/A	N/A	1115 (26.4)	158 (25.0)	0.93 (0.76-1.12)	.43	N/A	N/A
GP remote consultation, n (%)		852 (67.7)	832 (73.4)	1.32 (1.10-1.57)	.002	N/A	N/A	2354 (55.8)	376 (59.4)	1.16 (0.98-1.37)	.09	N/A	N/A
Admitted to hospital without COVID-19 symptoms, n (%)		82 (6.5)	112 (9.9)	1.57 (1.17-2.12)	.003	N/A	N/A	155 (3.7)	35 (5.5)	1.53 (1.04-2.21)	.03	N/A	N/A
Admitted to hospital with COVID-19 symptoms, n (%)		7 (0.6)	18 (1.6)	2.89 (1.25-7.45)	.02	N/A	N/A	14 (0.3)	<5^e^	1.43 (0.33-4.39)	.58	N/A	N/A
Hospital clinic in-person, n (%)		605 (48.1)	578 (51.0)	1.12 (0.96-1.32)	.15	N/A	N/A	1140 (27.0)	167 (26.4)	0.97 (0.80-1.17)	.73	N/A	N/A
Hospital clinic remote, n (%)		824 (65.5)	808 (71.3)	1.31 (1.10-1.56)	.002	N/A	N/A	1788 (42.4)	303 (47.9)	1.25 (1.05-1.47)	.01	N/A	N/A
COVID-19–positive result, n (%)		21 (1.7)	37 (3.3)	1.99 (1.17-3.47)	.01	N/A	N/A	82 (1.9)	15 (2.4)	1.22 (0.67-2.07)	.48	N/A	N/A
Persistent shielding^f^, n (%)		N/A	202 (25.2)	N/A	N/A	N/A	N/A	N/A	67 (14.4)	N/A	N/A	N/A	N/A
COVID-19 symptoms, n (%)		352 (28.0)	368 (32.5)	1.24 (1.04-1.48)	.02	N/A	N/A	995 (23.6)	163 (25.8)	1.12 (0.92-1.36)	.24	N/A	N/A
Development of COVID-19 symptoms over the 17 weeks in those without symptoms at baseline, n (%)		216 (17.6)	231 (21.1)	1.26 (1.02-1.55)	.03	N/A	N/A	611 (14.7)	77 (12.5)	0.83 (0.64-1.06)	.15	N/A	N/A

^a^CEV: clinically extremely vulnerable.

^b^Odds ratios (ORs) represent likelihood of adopting shielding behavior, expressed with associated 95% CI.

^c^N/A: not applicable.

^d^GP: general practitioner.

^e^We state “<5” where there are five or fewer patients within the criteria in question in order to preserve patient anonymity.

^f^Persistent shielding defined as shielding at baseline, week 9, and week 15.

**Table 3:** Patient characteristics and questionnaire responses grouped by clinically extremely vulnerable qualifying criteria.

CEV^a^ qualifying criteria	CEV, not shielding (N=1258), n (%)	CEV, shielding (N=1133), n (%)	OR^b^ (95% CI) (univariable)	*P* value
Solid-organ transplant	82 (6.5)	188 (16.6)	2.85 (2.18-3.77)	<.001
Cancer (active chemotherapy)	38 (3.0)	42 (3.7)	1.24 (0.79-1.94)	.35
Cancer (lung, active radiotherapy)	<5^c^	<5	1.11 (0.21-6.01)	.90
Cancer (blood/bone)	76 (6.0)	84 (7.4)	1.25 (0.90-1.72)	.18
Cancer (immunotherapy)	83 (6.6)	57 (5.0)	0.75 (0.53-1.06)	.10
Bone marrow transplant	<5	7 (0.6)	1.95 (0.59-7.46)	.29
Severe respiratory illness	156 (12.4)	212 (18.7)	1.63 (1.30-2.04)	<.001
Rare disease	77 (6.1)	97 (8.6)	1.44 (1.05-1.96)	.02
Immunosuppression	380 (30.2)	372 (32.8)	1.13 (0.95-1.34)	.17
Down syndrome^d^	N/A^e^	<5	N/A	N/A
Chronic kidney disease (on dialysis)^d^	32 (2.5)	17 (1.5)	0.58 (0.32-1.04)	.08
Pregnancy with heart disease	<5	N/A	N/A	N/A
Expert clinical advice^f^	445 (35.4)	285 (25.2)	0.61 (0.51-0.73)	<.001

^a^CEV: clinically extremely vulnerable.

^b^Odds ratios (ORs) represent likelihood of adopting shielding behavior, expressed with associated 95% CI.

^c^We state “<5” where there are five or fewer patients within the criteria in question in order to preserve patient anonymity.

^d^Clinical conditions appended to the original CEV list after week 2.

^e^N/A: not applicable.

^f^Expert clinical advice refers to the patient receiving advice to shield despite not being a member of an at-risk group according to the CEV criteria.

### Associations With Adopting Shielding Behavior

For CEV participants, multivariable analysis showed an independent association between shielding and being female, being Asian, an increase in yearly age, and receiving a letter advising the participant to shield. Similar associations were observed in the non-CEV group. Among CEV participants, living in a home with outdoor space was significant only in univariable analysis, but it was maintained in multivariable analysis in the non-CEV group. Likewise, being in a household consisting of three members (compared to a household with one member) was positively associated with shielding behavior in the univariable analysis in both the CEV and non-CEV groups; however, in the multivariable analysis, the CIs crossed 1 for the CEV group but did not in the non-CEV group. Overall, 30.5% of participants were CEV on the basis of clinical judgement rather than specific morbidity, and such an attribution was associated with being less likely to shield. Among qualifying CEV conditions, adopting shielding behavior was associated with being a solid organ transplant recipient, or having a severe respiratory illness or rare disease (Table 3).

The persistence of shielding behavior was more prevalent in the CEV group than in the non-CEV group (202/802, 25.2% vs 67/464, 14.4%). Receipt of a government letter advising shielding was reported in 3.7% (180/4849) of non-CEV participants and was associated with adoption of shielding behavior (OR 2.88, 95% CI 2.04-3.99).

During the study period, the total number of participants who tested positive for COVID-19 was low in both the CEV (n=58, 2.4%) and non-CEV (n=97, 4.4%) cohorts. In the CEV group, there was a significant association between shielding behavior and testing positive for COVID-19 (OR 1.99, 95% CI 1.17-3.47). In the non-CEV group, there was no significant association between shielding behavior and testing positive for COVID-19. The subjective reporting of new symptoms attributable to COVID-19 infection was associated with shielding behavior in the CEV group, with no difference observed between those adopting shielding and those not adopting shielding in the non-CEV group.

### Longitudinal Associations Between Shielding and Well-being

**Figure 2:**
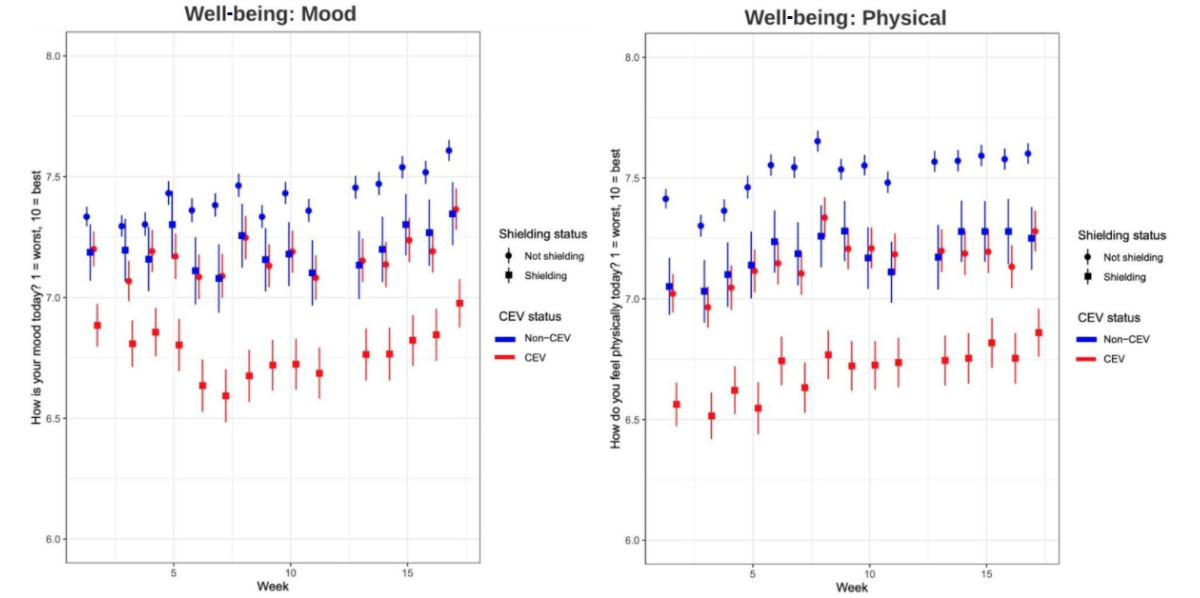
Longitudinal (17 weeks; April 9, 2020, to July 31, 2020) trends showing changes related to mood and physical well-being. CEV: clinically extremely vulnerable.

Longitudinal analysis of physical and mental well-being is displayed in Figure 2. Mental and physical well-being showed a quadratic relationship with time across the 17 weeks in both CEV and non-CEV patients (Multimedia Appendix 2 and Multimedia Appendix 3). In those who were CEV, shielding at baseline was associated with a lower rating of mental well-being (adjusted β −0.40, 95% CI −0.26 to −0.55) and physical well-being (adjusted β −0.51, 95% CI −0.37 to −0.66). Similar results were found for non-CEV patients (mental well-being: adjusted β −0.23, 95% CI −0.35 to −0.10; physical well-being: adjusted β −0.34, 95% CI −0.21 to −0.47). Unadjusted and adjusted results, along with coefficients for all variables included in the model, can be found in Multimedia Appendix 2 and Multimedia Appendix 3.

### Thematic Analysis of the Most Challenging Elements of Lockdown

**Figure 3:**
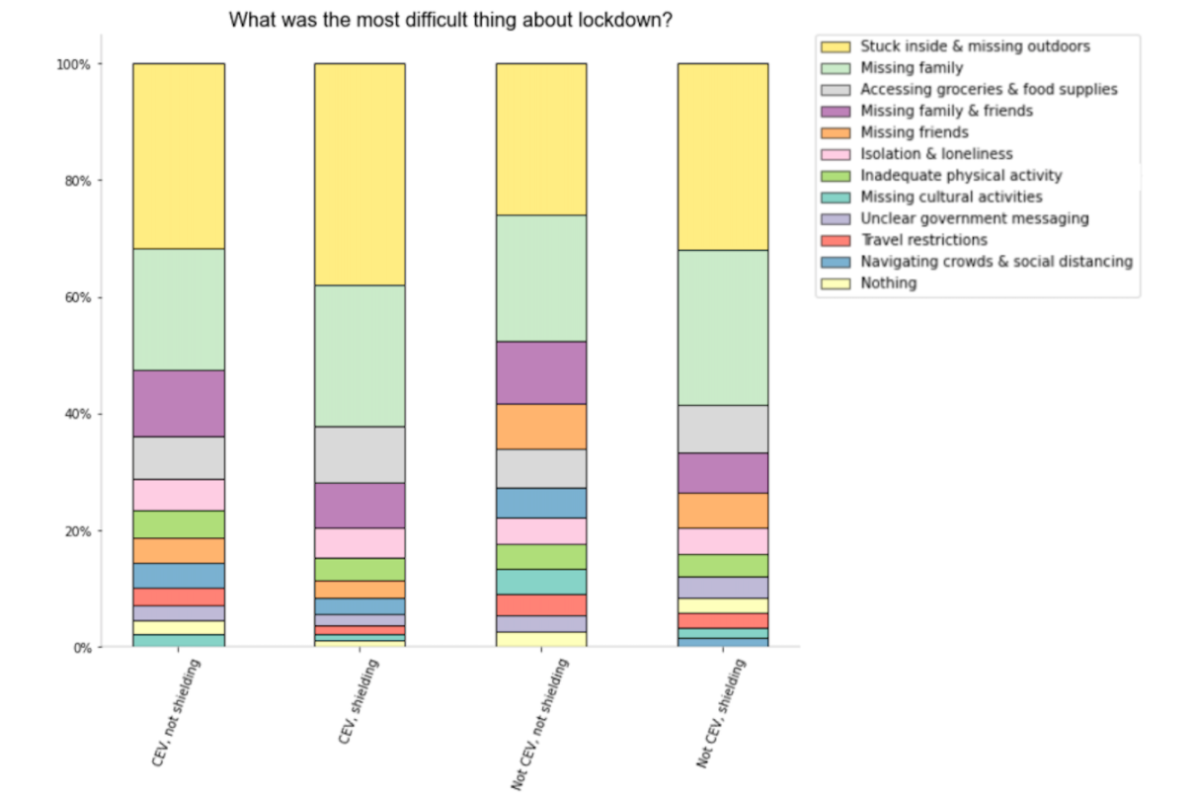
Week 14 questionnaire item posing a free-text question on the "most difficult thing about lockdown" (N=6300). CEV: clinically extremely vulnerable.

Across all groups, the most frequently occurring theme was feeling “stuck inside & missing outdoors,” followed by “missing family” (Figure 3). Prevalent in all four groups was the concern around accessing food and grocery supplies.

### Concern for the Need to Shield Again

**Figure 4:**
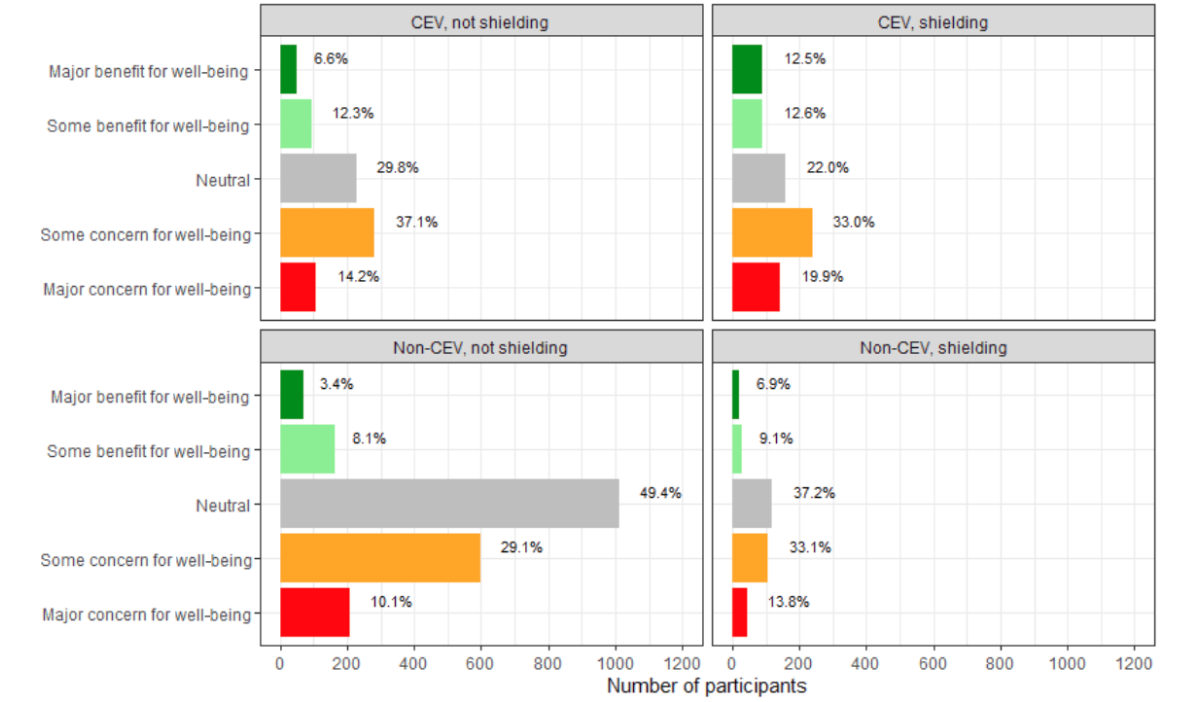
Week 29 questionnaire item measuring the level of concern for well-being if, in anticipation of a second UK national lockdown, advice is to assume shielding again (N=3818). CEV: clinically extremely vulnerable.

Across all groups, responses were skewed toward “some” or “major” concern for well-being should the requirement for further shielding arise (Figure 4). In the CEV group that adopted shielding behavior, 25.1% of participants projected that a requirement to shield again in anticipation of a second national lockdown would benefit their well-being, compared with 18.9% among those who did not adopt shielding behavior (*P*<.001).

## Discussion

### Main Findings

This longitudinal study of over 7000 NHS patients measured the determinants of shielding behavior and the impact on well-being throughout the first wave (17 weeks) of the UK’s COVID-19 pandemic. We found that approximately half of our sample’s CEV patients adopted shielding behavior (“staying at home at all times”) as per the advice at the time. This behavior was also reported by 15% of non-CEV participants. Our findings highlight that shielding behavior was associated with worse mental and physical well-being in both CEV and non-CEV cohorts, suggesting that the adoption of such behavior when not indicated may have resulted in avoidable detriments to physical and mental health.

The percentage of the UK public with “high anxiety” over COVID-19 peaked during the time of the week 1 questionnaire [14], which may explain why many decided to assume the behavior of being a “shielder,” despite not meeting the criteria. A survey of patients with arthritis who reported they were shielding (many of whom were non-CEV) suggested only 25% had received a government letter advising them to do so [15]. The timing of the baseline questionnaires also aligned with the first widespread reports that certain ethnic minorities were overrepresented among COVID-19 deaths [16,17], possibly explaining our observed positive association between shielding behavior and Asian ethnicity.

We found that only 50% of our sample’s CEV population reported receiving a government letter. This is of concern given our observed positive association between letter receipt and adoption of shielding behavior. Though such letters may empower patients to make informed decisions about shielding, our study also suggests some misattributions of CEV status, where subsequent receipt of advice to shield was associated with unnecessary adoption of shielding behavior. Conversely, it has also been reported that some patients initially advised to shield were later informed by text message of no longer needing to do so, fueling uncertainty on what advice to follow. The positive association with increasing age reflects widespread misunderstanding about who shielding advice applied to, with some headlines calling on the government to “set free” healthy individuals aged over 70 years [18].

The hyperinflammatory features of severe COVID-19 have led to the suggestion that some CEV patients are in fact protected by virtue of taking immunosuppressants, for example, oral steroids for rheumatoid arthritis [19], and that following the advice given to the general population may have been adequate. Early epidemiological descriptions of COVID-19 identified underrepresentation of people with chronic respiratory disease [20], with subsequent studies suggesting a protective effect from inhaled corticosteroid therapy [20-22]. Here, our findings indicate the possibility of confounding by behavior, namely that those with severe respiratory illness were among the most likely to shield during the early stages of the pandemic, allowing the first inference that shielding may have had a protective effect in this group.

The early months of the pandemic also exposed substantial inequalities and insecurities in food supply [23,24], with results from our qualitative analysis suggesting this was particularly marked among those shielding. Despite this, in the run up to the second national lockdown, a quarter of those CEV participants who originally shielded indicated “some” or “major” anticipated benefit to their well-being if further shielding was advised, suggesting negative impacts were far from universal.

Our findings are best interpreted in the context of their limitations. Though the LoC-19 participant cohort is large and uniquely rich (compared with less timely surveys on shielding from the Office of National Statistics [25]), covers the entire United Kingdom, and includes the spectrum of CEV patients, it is not fully representative of the general NHS patient population, particularly those traditionally underrepresented (eg, ethnic minorities). Notably, the digital divide that historically excludes older participants when using online survey tools for epidemiology studies is not represented here. However, the generalizability of the results is limited by this population having elected in the first instance to monitor their well-being using the provided tool. Participants completed, on average, 75% of the questionnaires in the first 17 weeks. Though this is a high response rate, our analyses did not account for potential biases from differential responses/retention rates on a weekly basis. As has been a common theme for COVID-19, the positive association with household outdoor space suggests the ability to shield (particularly for non-CEV individuals) may also follow a social gradient. Our study did not capture all possible social determinants, such as the need to sustain income and provide care to others [26]. Validated multiquestion instruments would have been preferable for evaluating mood and physical well-being; however, these were less suitable for this longitudinal study owing to the risk of biases, including responder fatigue. CEV status was captured at week 31 and not by baseline (week 1 or 2) questionnaires. The CEV list went through several iterations, and conditions, such as Down syndrome and chronic kidney disease on dialysis, were added later [27], such that recording CEV status in week 31 enabled comprehensive capture. However, this resulted in a lower number of participants eligible for inclusion in the analysis. Recall bias is unlikely to have been an issue for capturing self-reported CEV status by qualifying the clinical condition in week 31, though this time point may explain our result that CEV status determined by clinical advice was associated with being less likely to shield. These participants may have been uninformed of their CEV status until several weeks/months into the pandemic and therefore were initially not shielding at receipt of the baseline questionnaires. All participants, regardless of CEV status or shielding behavior, completed >75% of the sequential weekly questionnaires. Our data set nonetheless contains a variable level of weekly nonresponse, somewhat ameliorated by the large sample size.

Our finding that overall shielding negatively impacted both mental and physical well-being has also been observed in small studies focusing on specific disease groups such as cystic fibrosis [28] and complex dermatology [29]. Nonetheless, the ongoing pandemic led to an improved understanding of what determines the highest risk from COVID-19, with subsequent development and adoption of predictive risk modeling [30] that identified and extended shielding advice to a further 1.7 million people during the third national lockdown (February 2021) [31]. However, this was not informed by evidence that shielding is any more protective than established advice for the rest of the population. The lack of community testing during the first months of the COVID-19 pandemic means we are unable to definitively comment on whether the act of shielding was associated with lower incidence of COVID-19. In fact, our results suggest both positive COVID-19 testing and new symptoms indicative of COVID-19 were positively associated with shielding behavior; however, this may also be related to, as we observed, more health and social care encounters in this group. Therefore, beyond intuition, evaluating whether the shielding policy of the first wave of the pandemic in the United Kingdom achieved its primary objective of reducing cases of COVID-19 will remain challenging, but this study gives the first indication that the act of shielding precipitated a trade-off with well-being, and that unclear messaging may have driven inconsistencies in how shielding behavior was adopted. Ultimately, a partnership needs to be achieved with those we believe are at increased risk from COVID-19 to empower them to reduce their risk through consistent communication, openness about uncertainty, and respect for personal autonomy.

### Conclusion

Future health policy must balance the as yet unproven benefit of shielding for protection from COVID-19 against our findings that shielding negatively impacted well-being and was adopted by many in whom it was not indicated and variably in whom it was indicated. Our findings highlight the need for clearer public health messaging and support for well-being if shielding is to be advised in future pandemic scenarios.

## Appendix

Multimedia Appendix 1 Question items included in Care Information Exchange questionnaires.

Multimedia Appendix 2 Mixed effects linear regression for the association between shielding and mood and physical rating (clinically extremely vulnerable).

Multimedia Appendix 3 Mixed effects linear regression for the association between shielding and mood and physical rating (nonclinically extremely vulnerable).

## Abbreviations

CEV: clinically extremely vulnerable
CIE: Care Information Exchange
NHS: National Health Service
OR: odds ratio
