# Supplementary material for: Determinants of Shielding Behavior During the COVID-19 Pandemic and Associations With Well-being Among National Health Service Patients: Longitudinal Observational Study
Source: JMIR Public Health Surveill. 2021 Sep 20;7(9):e30460. doi: 10.2196/30460 (PMC8454693; doi:10.2196/30460)
Supplement: Multimedia Appendix 1 [file publichealth_v7i9e30460_app1.docx]

**Multimedia Appendix 1.** Question items included in Care Information Exchange questionnaires.

| **Question stem** | **Answer options** |
| --- | --- |
| How is your mood today? | 1 = worst  2  3  4  5  6  7  8  9  10 = best |
| How do you feel physically today? | 1 = worst  2  3  4  5  6  7  8  9  10 = best |
| Have you or anyone in your household received a letter or message informing you that you are in the population at ‘high risk’ from coronavirus? Please tick all that apply. | No - Neither myself or anyone in my household is at 'high risk'  No - but I think I should have  No - but someone in my household is at 'high risk'  Yes - letter about me  Yes - letter about someone in my household |
| Do you have a cough? | No  Yes -- I have a new, dry cough  Yes -- I have a new cough bringing up phlegm  Yes -- but I usually have a cough like this Yes -- my usual cough has worsened |
| Are you experiencing unusual shortness of breath compared to what's normal for you? | No  Yes -- Mild symptoms, slight shortness of breath during ordinary activity  Yes – Significant symptoms, breathing is comfortable only at rest  Yes -- Severe symptoms, breathing difficult even at rest |
| Have you had a fever and did you take your temperature in the last week? | I have NOT felt feverish  I have felt feverish but did not check my temperature  I felt feverish and my temperature was BELOW 38 degrees Celcius  I felt feverish and my temperature measured ABOVE 38 degrees Celcius |
| Have you experienced any of these other symptoms? Please tick all that apply | I haven't had any new symptoms  Loss of sense of smell  Loss of appetite (skipping meals)  Diarrhoea  Vomiting  Fatigue  Sneezing  Chest pain / tightness  Sore throat  Runny nose  Itchy eyes  Headache  Joint pain / aches |
| Does anyone in your household have a new cough or fever? | Not applicable  No  Yes |
| Including yourself, how many people live in your household? | 1  2  3  4  5  6  7  8 |
| Have you or anyone in your house been tested for coronavirus? Please tick all that apply | No testing  I was tested - positive result  I was tested - awaiting result  I was tested - negative result  Household member tested - positive result  Household member tested - awaiting result  Household member tested - negative result |
| Have you had any healthcare contact since the lockdown started? Please tick all that apply | No  Yes - remote appointment with my GP (phone/video)  Yes - I attended my GP practice for an appointment  Yes - remote appointment with hospital (phone/video)  Yes - I attended hospital for an appointment  Yes - attended Accident and Emergency  Yes - I was admitted to hospital (not because of coronavirus)  Yes - I was admitted to hospital with symptoms of coronavirus |
| PLEASE TICK ANY OF THESE THAT APPLY TO YOU: | Solid organ transplant recipient  Cancer undergoing active chemotherapy  Lung cancer undergoing radical radiotherapy  Cancer of the blood or bone marrow such as leukaemia, lymphoma or myeloma at any stage of treatment  Cancer and receiving immunotherapy or other treatments that you have been told can affect the immune system  Bone marrow or stem cell transplants in the last 6 months or still taking immunosuppressive drugs  Severe respiratory conditions including all cystic fibrosis, severe asthma and severe chronic obstructive pulmonary disease (COPD) - (please note - only if what you would consider SEVERE)  Rare diseases that you have been advised significantly increase the risk of infections  Taking immunosuppression therapies for any condition that you have been told increase risk of infection  Adult with Down’s syndrome  Adult on dialysis OR with severe chronic kidney disease (stage 5)  Pregnant with significant heart disease  Advised I am ‘clinically extremely vulnerable’ by my GP/doctor based on judgement rather than any of the above |
| On balance, how has your previous experience of lockdown affected your view of shielding or need to shield again? | Major concern for wellbeing  Some concern for wellbeing  Neutral  Some benefit for wellbeing  Major benefit for wellbeing |
