# Supplementary material for: Determinants of Shielding Behavior During the COVID-19 Pandemic and Associations With Well-being Among National Health Service Patients: Longitudinal Observational Study
Source: JMIR Public Health Surveill. 2021 Sep 20;7(9):e30460. doi: 10.2196/30460 (PMC8454693; doi:10.2196/30460)
Supplement: Multimedia Appendix 2 [file publichealth_v7i9e30460_app2.docx]

**Multimedia Appendix 2.** Mixed effects linear regression for the association between shielding and mood and physical rating (clinically extremely vulnerable).

| Model | Variable | Beta coefficients for mood | Beta coefficients for physical |
| --- | --- | --- | --- |
| Null model | Intercept | 6.894 (6.818 to 6.969) | 6.949 (6.876 to 7.022) |
|  | Week | 0.002 (-0.002 to 0.007) | 0.016 (0.012 to 0.020) |
|  | Week² | 0.003 (0.002 to 0.004) | -0.001 (-0.002 to -0.001) |
| Unadjusted model | Intercept | 7.047 (6.950 to 7.144) | 7.156 (7.060 to 7.252) |
|  | Week | 0.002 (-0.002 to 0.007) | 0.016 (0.012 to 0.020) |
|  | Week² | 0.003 (0.002 to 0.004) | -0.001 (-0.002 to -0.001) |
|  | Shielding | -0.323 (-0.452 to -0.194) | -0.436 (-0.569 to -0.304) |
| Adjusted model | Intercept | 6.035 (5.604 to 6.467) | 6.268 (5.825 to 6.710) |
|  | Week | 0.004 (-0.000 to 0.008) | 0.017 (0.013 to 0.021) |
|  | Week² | 0.003 (0.002 to 0.004) | -0.002 (-0.002 to -0.001) |
|  | Shielding | -0.402 (-0.546 to -0.257) | -0.514 (-0.662 to -0.365) |
|  | Age (+1 year) | 0.016 (0.010 to 0.022) | 0.014 (0.008 to 0.020) |
|  | Female | -0.261 (-0.402 to -0.120) | -0.245 (-0.390 to -0.100) |
|  | Ethnicity: White | - | - |
|  | Ethnicity: Asian | -0.180 (-0.418 to 0.057) | -0.179 (-0.422 to 0.065) |
|  | Ethnicity: Black | -0.081 (-0.436 to 0.274) | -0.208 (-0.573 to 0.157) |
|  | Ethnicity: Mixed | -0.155 (-0.836 to 0.526) | -0.128 (-0.827 to 0.571) |
|  | Ethnicity: Other | -0.485 (-0.798 to -0.172) | -0.539 (-0.860 to -0.218) |
|  | Key worker | 0.047 (-0.180 to 0.274) | 0.028 (-0.205 to 0.261) |
|  | Outdoor space | 0.214 (0.019 to 0.409) | 0.170 (-0.030 to 0.370) |
|  | Smoking status: Non-smoker | - | - |
|  | Smoking status: Ex-smoker | -0.282 (-0.429 to -0.134) | -0.266 (-0.417 to -0.114) |
|  | Smoking status: Smoker | -0.643 (-0.942 to -0.344) | -0.528 (-0.835 to -0.221) |
|  | Household number: 1 | - | - |
|  | Household number: 2 | 0.401 (0.226 to 0.576) | 0.392 (0.212 to 0.571) |
|  | Household number: 3 | 0.127 (-0.097 to 0.352) | 0.081 (-0.149 to 0.312) |
|  | Household number: 4 | 0.364 (0.094 to 0.633) | 0.310 (0.033 to 0.587) |
|  | Household number: 5+ | 0.337 (0.007 to 0.668) | 0.354 (0.015 to 0.693) |
|  | Receipt of risk letter | 0.099 (-0.044 to 0.241) | 0.144 (-0.002 to 0.290) |

Null model and unadjusted model N = 2,391, adjusted model N = 1,992
