# Supplementary material for: Determinants of Shielding Behavior During the COVID-19 Pandemic and Associations With Well-being Among National Health Service Patients: Longitudinal Observational Study
Source: JMIR Public Health Surveill. 2021 Sep 20;7(9):e30460. doi: 10.2196/30460 (PMC8454693; doi:10.2196/30460)
Supplement: Multimedia Appendix 3 [file publichealth_v7i9e30460_app3.docx]

**Multimedia Appendix 3.** Mixed effects linear regression for the association between shielding and mood and physical rating (non-clinically extremely vulnerable).

| Model | Variable | Beta coefficients for mood | Beta coefficients for physical |
| --- | --- | --- | --- |
| Null model | Intercept | 7.331 (7.285 to 7.377) | 7.482 (7.439 to 7.526) |
|  | Week | 0.013 (0.010 to 0.015) | 0.013 (0.011 to 0.016) |
|  | Week² | 0.001 (0.001 to 0.002) | -0.001 (-0.002 to -0.001) |
| Unadjusted model | Intercept | 7.354 (7.306 to 7.403) | 7.523 (7.477 to 7.569) |
|  | Week | 0.013 (0.010 to 0.015) | 0.013 (0.011 to 0.016) |
|  | Week² | 0.001 (0.001 to 0.002) | -0.001 (-0.002 to -0.001) |
|  | Shielding | -0.177 (-0.297 to -0.058) | -0.310 (-0.428 to -0.192) |
| Adjusted model | Intercept | 6.174 (5.920 to 6.427) | 6.519 (6.268 to 6.769) |
|  | Week | 0.013 (0.010 to 0.015) | 0.013 (0.011 to 0.016) |
|  | Week² | 0.001 (0.001 to 0.002) | -0.001 (-0.002 to -0.001) |
|  | Shielding | -0.225 (-0.353 to -0.097) | -0.339 (-0.466 to -0.212) |
|  | Age (+1 year) | 0.016 (0.012 to 0.019) | 0.013 (0.010 to 0.017) |
|  | Female | -0.147 (-0.235 to -0.059) | -0.103 (-0.190 to -0.016) |
|  | Ethnicity: White | - | - |
|  | Ethnicity: Asian | -0.160 (-0.333 to 0.013) | -0.196 (-0.368 to -0.025) |
|  | Ethnicity: Black | -0.131 (-0.398 to 0.135) | -0.169 (-0.434 to 0.095) |
|  | Ethnicity: Mixed | -0.396 (-0.721 to -0.070) | -0.469 (-0.792 to -0.146) |
|  | Ethnicity: Other | -0.060 (-0.260 to 0.140) | -0.100 (-0.298 to 0.098) |
|  | Key worker | -0.000 (-0.132 to 0.132) | -0.058 (-0.188 to 0.073) |
|  | Outdoor space | 0.386 (0.270 to 0.501) | 0.345 (0.230 to 0.459) |
|  | Smoking status: Non-smoker | - | - |
|  | Smoking status: Ex-smoker | -0.120 (-0.212 to -0.028) | -0.086 (-0.177 to 0.006) |
|  | Smoking status: Smoker | -0.404 (-0.586 to -0.221) | -0.382 (-0.562 to -0.201) |
|  | Household number: 1 | - | - |
|  | Household number: 2 | 0.234 (0.125 to 0.343) | 0.202 (0.093 to 0.310) |
|  | Household number: 3 | 0.121 (-0.020 to 0.262) | 0.056 (-0.084 to 0.196) |
|  | Household number: 4 | 0.184 (0.021 to 0.347) | 0.136 (-0.026 to 0.297) |
|  | Household number: 5+ | 0.151 (-0.064 to 0.365) | 0.140 (-0.073 to 0.353) |
|  | Receipt of risk letter | 0.033 (-0.201 to 0.266) | 0.032 (-0.200 to 0.263) |

Null model and unadjusted model N = 4,848, adjusted model N = 4,140
